# A novel method to fabricate CoFe_2_O_4_/SrFe_12_O_19_ composite ferrite nanofibers with enhanced exchange coupling effect

**DOI:** 10.1186/s11671-015-0829-z

**Published:** 2015-03-14

**Authors:** Lining Pan, Derang Cao, Panpan Jing, Jianbo Wang, Qingfang Liu

**Affiliations:** Key Laboratory for Magnetic and Magnetic Materials of the Ministry of Education, Lanzhou University, Lanzhou, 730000 People’s Republic of China

**Keywords:** Cobalt powder, CoFe_2_O_4_/SrFe_12_O_19_ nanocomposite nanofibers, Exchange coupling interaction, Crystallite size

## Abstract

Nanocomposite of CoFe_2_O_4_/SrFe_12_O_19_ has been synthesized by the electrospinning and calcination process. A novel method that cobalt powder was used to replace traditional cobalt salt in the precursor sol-gel for electrospinning was proposed. The crystal structures, morphologies, and magnetic properties of these samples have been characterized in detail. Moreover, when the average crystallite size of the hard/soft phases reached up to an optimal value, the CoFe_2_O_4_ have an enhanced saturation magnetization of 62.8 emu/g and a coercivity of 2,290 Oe. Significantly, the hysteresis loops for the nanocomposites show a single-phase magnetization behavior, and it has been found that the exchange coupling interaction strongly exists in the CoFe_2_O_4_/SrFe_12_O_19_ magnetic nanocomposite nanofibers.

## Background

Cobalt ferrite (CoFe_2_O_4_) has attracted enormous concern due to its significant properties such as remarkable chemical and thermal stabilities, good mechanical hardness, and high saturation magnetization (*M*_s_). They have been widely applied in diverse fields, including magnetic resonance imaging, magnetically targeted drug, permanent magnets, microwave absorption, and magnetic data storage [1-4]. Recently, a number of studies about the synthesis of CoFe_2_O_4_ have been reported. CoFe_2_O_4_ nanoparticles were fabricated by a sol-gel method by Xi et al. [5], Mazario fabricated the CoFe_2_O_4_ ferrite nanoparticles by an electrochemical method [6], CoFe_2_O_4_ nanofibers was obtained by the electrospinning in 2009 [7], Liu et al. prepared the CoFe_2_O_4_ nanoplatelets using a facile hydrothermal route [8], and so on. However, the applications of CoFe_2_O_4_ are often limited by its relatively low coercivity (*H*_c_). The multitudinous experimental results show that the *H*_c_ could be improved drastically combining CoFe_2_O_4_ with hard ferrites to form nanocomposite via exchange coupling effect [9-14]. Among the hard ferrites, strontium hexaferrite (SrFe_12_O_19_) has significant performances such as high Curie temperature (*T*_c_), high *M*_s_, high *H*_c_, large magnetocrystalline anisotropy (*H*_*k*_), and excellent chemical stability [15-17]. So far, the growing efforts have been devoted to the investigation of nanocomposites of CoFe_2_O_4_/SrFe_12_O_19_ [18,19]. Nevertheless, these investigations indicated the presence of impurities such as α-Fe_2_O_3_ [19,20], which exhibited a twisted hysteresis loop and an incomplete exchange coupling between the hard and soft phases [9,13]. Furthermore, the presence of incomplete exchange coupling behavior severely leads to the decrease of magnetic properties. It is important to obtain the samples with no impurities and strong exchange coupling interaction. Compared with the former methods, electrospinning has been certified to be a simple, low-cost, and versatile technique capable of generating numerous one-dimensional (1D) nanostructures. As one of the typical 1D nanostructures, nanofibers have high surface-to-volume ratio, high aspect ratio, and big shape anisotropy compared to nanoparticle and bulk [19,20]. Therefore, plenty of interests have been focused on the nanofibers because of their novel properties and potential applications [11,21].

Originally, when the cobalt salt was used in the synthetic process, cobalt ion replaces easily Fe or Sr ion or both in the SrFe_12_O_19_ [22]. Because the substitution between the ions leads to the destruction of the ratio of solution, the α-Fe_2_O_3_, SrFe_2_O_4_, and other impurity phases are easy to form. And to avoid the substitution, we proposed a novel way that cobalt powder was used in the precursor sol-gel to form a suspending liquid for electrospinning. In this work, the CoFe_2_O_4_/SrFe_12_O_19_ nanofibers were obtained by electrospinning followed by heating treatment. And the structure, chemical component, and magnetic properties were characterized in detail. The hard/soft phases contacted closer sufficiently than traditional composite particles and previous nanofibers, and the crystallite size of the two phases also achieved an optimal value, which lead to a strong exchange coupling.

## Methods

**Figure 1:**
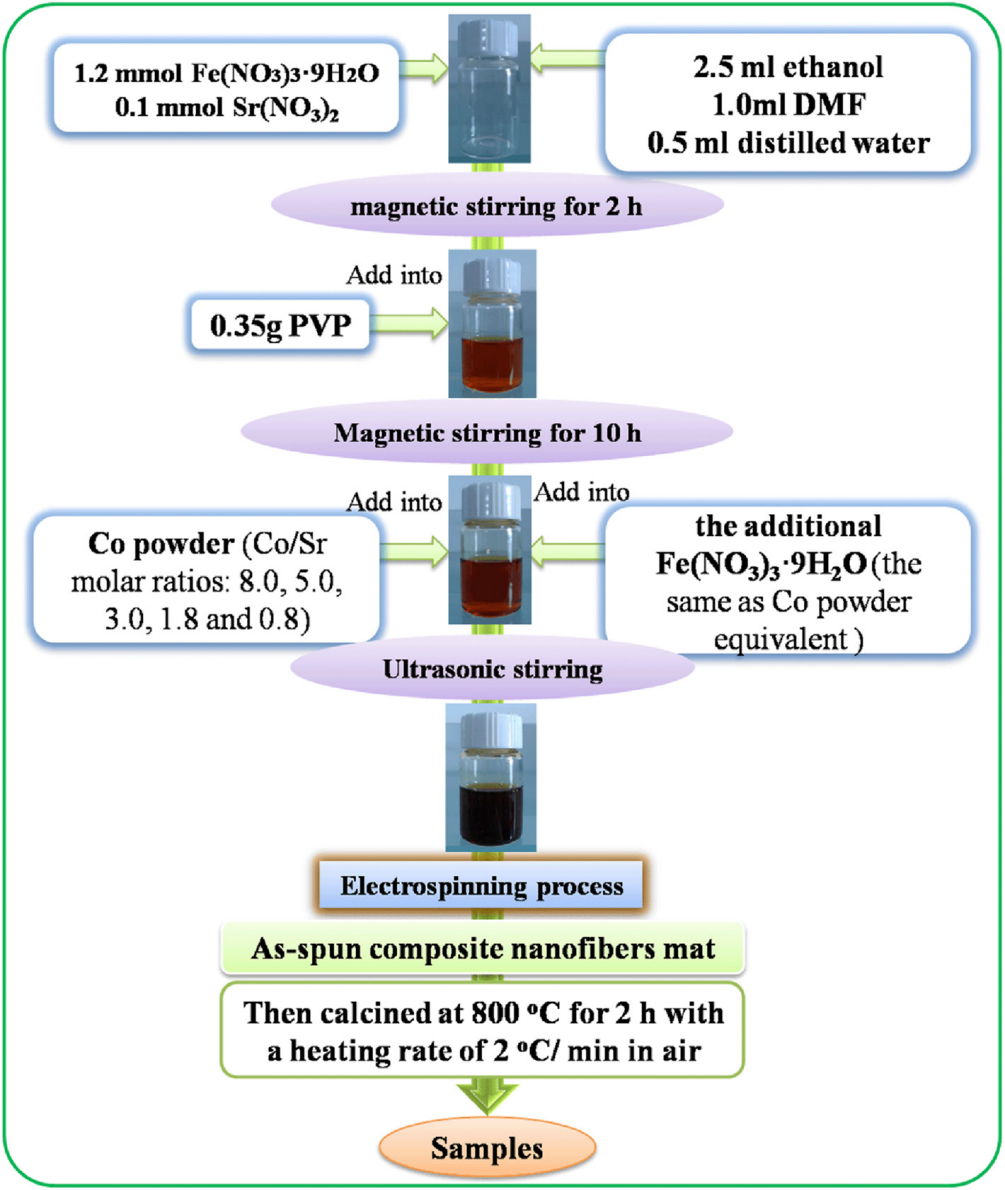
**The graph of experimental flow.**

In this work, CoFe_2_O_4_/ SrFe_12_O_19_ composite nanofibres were synthesized by electrospinning combined with the sol-gel technique. All chemical reagents were of chemical grade. As shown in Figure 1, in the typical synthesis, 1.2 mmol Fe(NO_3_)_3_ · 9H_2_O and 0.1 mmol Sr(NO_3_)_2_ were dissolved together in a mixed solvent of 0.5 ml deionized water, 1.0 ml *N*,*N*-dimethylformamide (DMF), and 2.5 ml ethanol (C_2_H_5_OH), and then followed by magnetic stirring for 2 h. Then, 0.35 g of poly (vinyl pyrrolidone) (PVP; *M*_w_ = 1,300,000) was dissolved in the above solution and stirred for 10 h at room temperature to form a homogeneous viscous solution. Subsequently, the Co powder (Sinopharm Chemical Reagent Co., Ltd., Shanghai, China, 99%, 200 mesh) was dispersed ultrasonically in the PVP/nitrate solution with different Co/Sr^2+^ molar ratios (8.0, 5.0, 3.0, 1.8, and 0.8). And the additional equivalent Fe(NO_3_)_3_ · 9H_2_O was also added into the above solution to form the resultant precursor. The electrospinning process was performed at a voltage of 13.5 kV DC, with a 15-cm spacing between the needle tip and the collector, and a feed rate of 0.3 ml/h was pumped by a syringe pump. Finally, the as-electrospun nanofibers were collected and annealed at 800°C for 2 h with a heating rate of 2°C/min in air and then cooled naturally to room temperature.

X-ray diffraction (XRD) patterns were recorded by a PANalytical diffractometer (PANalytical B.V., Almelo, The Netherlands) using Cu Kα radiation. Field emission scanning electron microscopy (FE-SEM; Hitachi S-4800, Hitachi, Ltd., Chiyoda, Tokyo, Japan) and transmission electron microscopy (TEM; Tecna™ G^2^F30, FEI Co., Hillsboro, OR, USA) equipped with energy-dispersive X-ray analysis (EDX; Oxford Instruments, Abingdon, Oxfordshire, UK) were used to characterize the microstructure of the samples. The magnetic hysteresis loops were measured by a vibrating sample magnetometer (VSM; MicroSense EV9, MicroSense, LLC, Lowell, MA, USA) at room temperature with a maximum applied field of 20 kOe.

## Results and discussion

**Figure 2:**
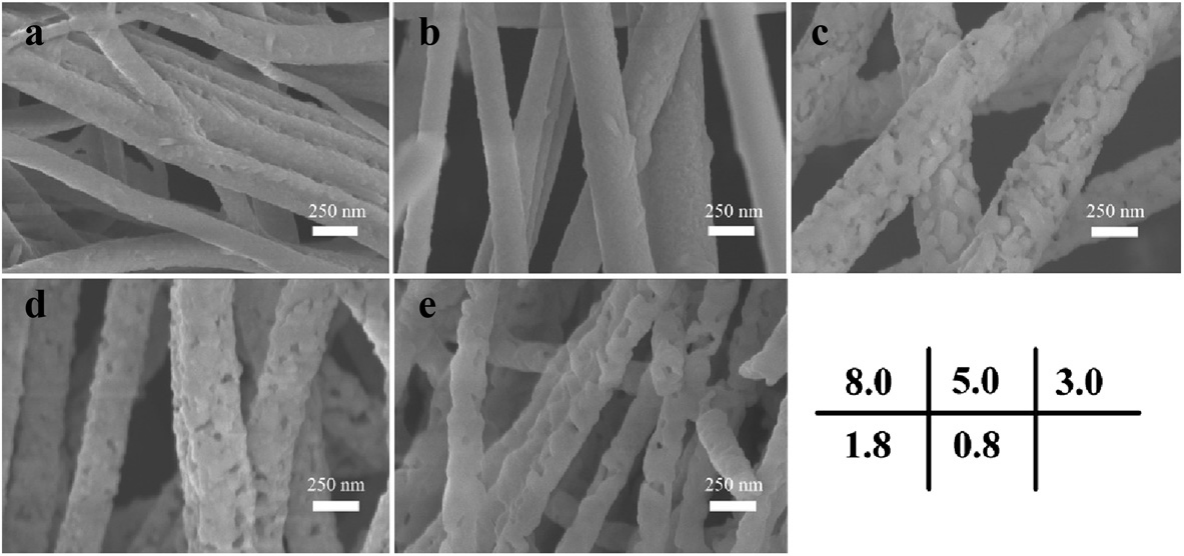
**SEM images of CoFe**_2_ **O**_4_ **/SrFe**_12_ **O**_19_ **nanofibers with different Co/Sr** ^2+^ **molar ratios: (a) 8.0, (b) 5.0, (c) 3.0, (d) 1.8, and (e) 0.8.**

As shown in Figure 2, SEM images clearly exhibit the morphologies of CoFe_2_O_4_/SrFe_12_O_19_ nanofibers with different Co/Sr^2+^ molar ratios (8.0, 5.0, 3.0, 1.8, and 0.8). Continuously linear structure and uniform diameter can be seen in all annealed nanofibers after the PVP was removed. And the diameter of these nanofibers ranged from 200 to 350 nm. When the Co/Sr^2+^ molar ratio is higher than 5.0, the nanofiber surface is smooth in Figure 2a,b. Decreasing the Co/Sr^2+^ molar ratio, the nanofibers possess a slightly rough surface and hollow structure. It indicated that the crystallite of the nanofibers grow up with the increase of Sr^2+^.

**Figure 3:**
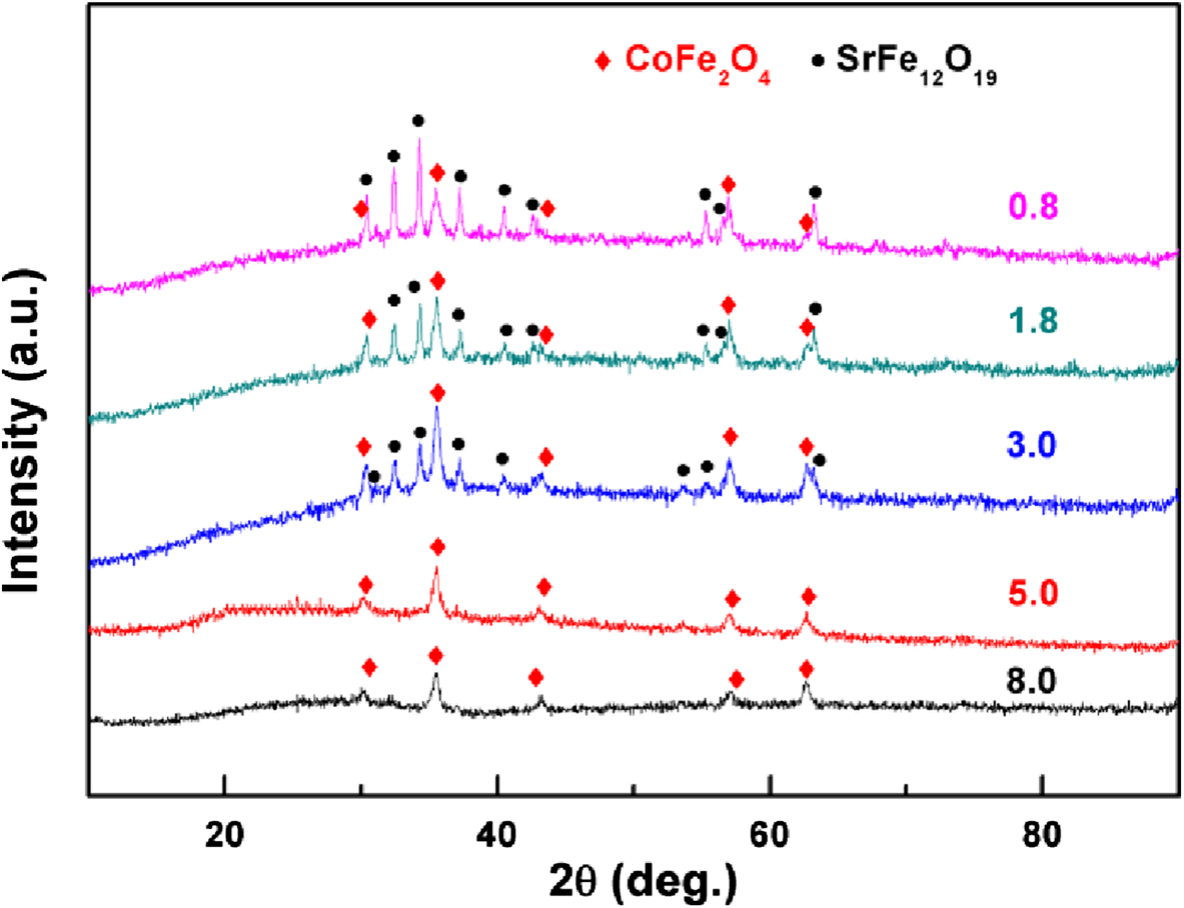
**XRD patterns of CoFe**_2_ **O**_4_ **/SrFe**_12_ **O**_19_ **nanofibers with different Co/Sr** ^2+^ **molar ratios.**

The crystal structure of the samples is investigated by XRD technique, and the patterns are shown in Figure 3. For the samples with Co/Sr^2+^ molar ratio of 8.0 and 5.0, only the diffraction peaks of CoFe_2_O_4_ rather than SrFe_12_O_19_ are found because the Sr^2+^ ratios in their precursors are low. It seems that the diffraction peaks of SrFe_12_O_19_ begin to appear when the Co/Sr^2+^ molar ratio is 3.0, and they become narrower and stronger with the advance of Sr^2+^ ions. When the Co/Sr^2+^ molar ratio is 1.8, the diffraction intensities of CoFe_2_O_4_ phase and SrFe_12_O_19_ phase are approximately equal. Furthermore, the content of CoFe_2_O_4_ reduces while that of SrFe_12_O_19_ increases with the decrease of Co/Sr^2+^ molar ratio. Regarding the formation of CoFe_2_O_4_/SrFe_12_O_19_ nanofibers, the possible chemical reactions during the sintering process are inferred as follows:

- Fe(NO_3_)_3_ → Fe_2_O_3_ + NO_*x*_
- Sr(NO_3_)_2_ → SrO + NO_*x*_
- SrO + CO_2_ → SrCO_3_
- SrCO_3_ + Fe_2_O_3_ → SrFe_2_O_4_ + CO_2_
- SrFe_2_O_4_ + 5Fe_2_O_3_ → SrFe_12_O_19_ [23]
- 2Co + O_2_ → 2CoO
- CoO + Fe_2_O_3_ → CoFe_2_O_4_ [18]

Furthermore, the average crystallite size of CoFe_2_O_4_ and SrFe_12_O_19_ were calculated using the Scherrer equation (*L* = *Kλ*/*β* cos *θ*, where *λ* is the X-ray wavelength in nanometer (nm), *β* is the full width at half maximum (FWHM) of diffraction peak, and *K* is a constant related to crystallite shape, normally taken as 0.89 [24]), and the values are listed in Table 1. With the Co/Sr^2+^ molar ratio changing from 3.0 to 0.8, the crystallite size of SrFe_12_O_19_ increases from 26 to 29 nm, but that of CoFe_2_O_4_ was basically equal to about 17 nm. Therefore, the increase of Sr^2+^ ions, meaning to increase the SrFe_12_O_19_ content, leads to the increase of the average crystallite size of the nanofiber.

**Table 1:** **Average crystallite size of the samples with different Co/Sr** ^2+^ **molar ratios**

**Molar ratio (Co/Sr** ^2+^ **)**	${\boldsymbol{D}}_{\mathbf{SrF}{\mathbf{e}}_{\mathbf{12}}{\mathbf{O}}_{\mathbf{19}}}$ **(nm)**	${\boldsymbol{D}}_{\mathbf{CoF}{\mathbf{e}}_{\mathbf{2}}{\mathbf{O}}_{\mathbf{4}}}$ **(nm)**
8.0	/	17
5.0	/	17
3.0	26	18
1.8	27	18
0.8	29	16

**Figure 4:**
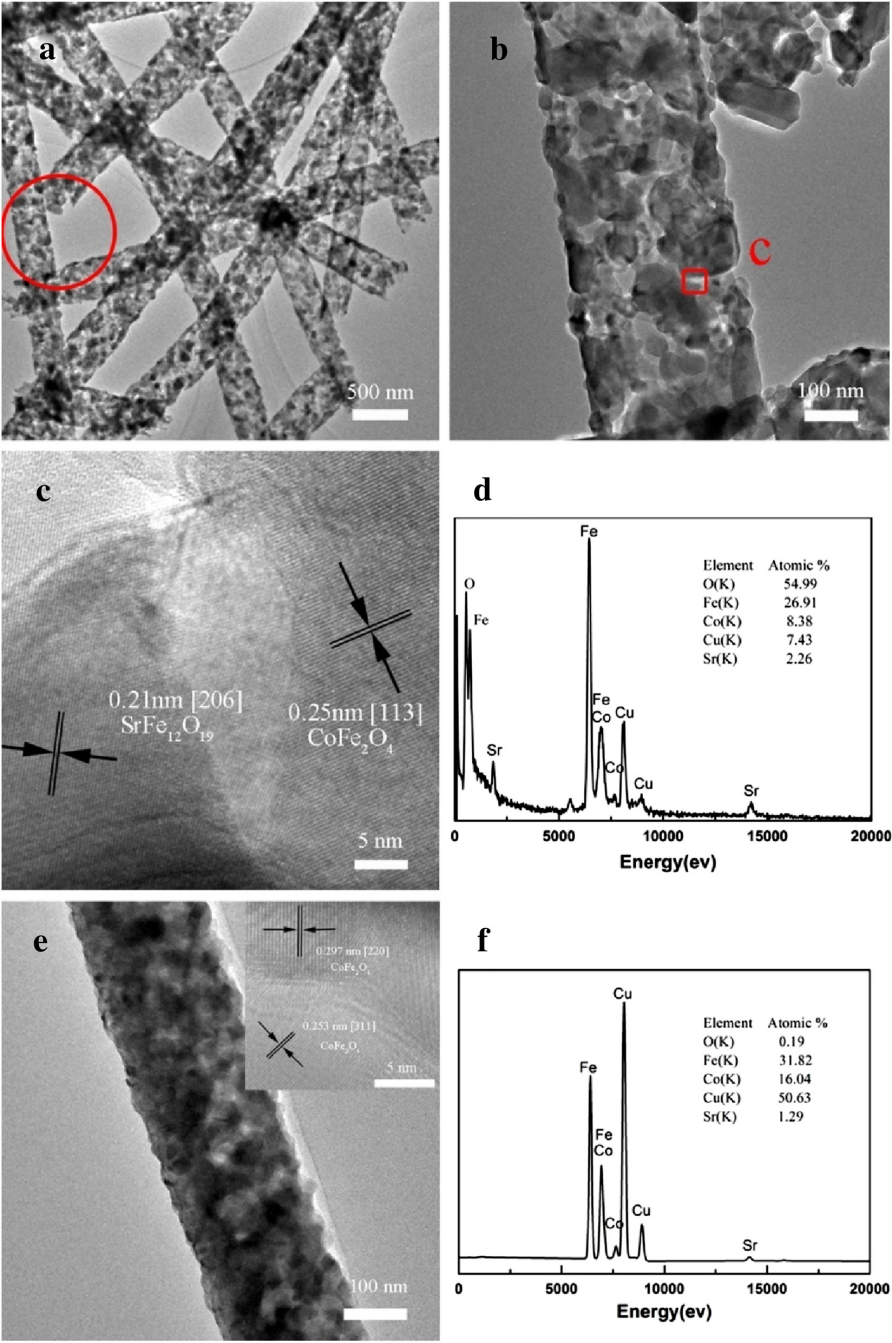
**TEM and HRTEM images and EDX spectrum of CoFe**_2_ **O**_4_ **/SrFe**_12_ **O**_19_ **nanofibers.** TEM images **(a, b)**, HRTEM image **(c)**, and EDX spectrum **(d)** of CoFe_2_O_4_/SrFe_12_O_19_ nanofibers with a Co/Sr^2+^ molar ratio of 1.8. TEM image **(e)**, top left inset in (e) showing a HRTEM pattern, and EDX spectrum **(f)** of CoFe_2_O_4_/SrFe_12_O_19_ nanofibers with a Co/Sr^2+^ molar ratio of 8.0.

The representative TEM images of the CoFe_2_O_4_/SrFe_12_O_19_ nanofibers with the Co/Sr^2+^ molar ratio of 1.8 are presented in Figure 4a,b; they show a continuously linear structure and uniform diameter, which is corresponding to the above SEM observation (Figure 2). It is easily seen that the nanofibers consisted of two sizes of grains: one looks like hexagonal plate type structure SrFe_12_O_19_ and another is cubical CoFe_2_O_4_. Contrasting to the result of the average crystallite size calculated by XRD, the crystallite size is smaller than the grain obtained by TEM, so we do not make sure that every grain is single crystalline. Compared with the sample in Figure 4b, the CoFe_2_O_4_/SrFe_12_O_19_ nanofibers with a Co/Sr^2+^ molar ratio of 8.0 in Figure 4e present a smoother surface, and the grains are smaller than the other samples. As shown in Figure 4e, the nanofibers are composed of uniform grains, and the grains densely stacked along the direction of nanofiber axis.

A high-resolution TEM image of the area is marked by red square in Figure 4b; as shown in Figure 4c, the interplanar spacing is measured to be 0.21 nm, which is consistent with the (206) crystallographic orientation of M-type hexagonal SrFe_12_O_19_. And another interplanar spacing is measured to be 0.25 nm, corresponding to the separation between the (113) lattice planes of CoFe_2_O_4_. The EDX spectrum of the nanofibers are shown in Figure 4d,f, confirming the presence of Fe, O, Sr, and Co in the samples, which is in good agreement with the mentioned result of XRD in Figure 3. So, it can be inferred that SrFe_12_O_19_ phase also exist in the samples with Co/Sr^2+^ molar ratio of 5.0 and 8.0. But the SrFe_12_O_19_ phase cannot be observed by corresponding XRD due to the tiny Sr element contents in these samples.

**Figure 5:**
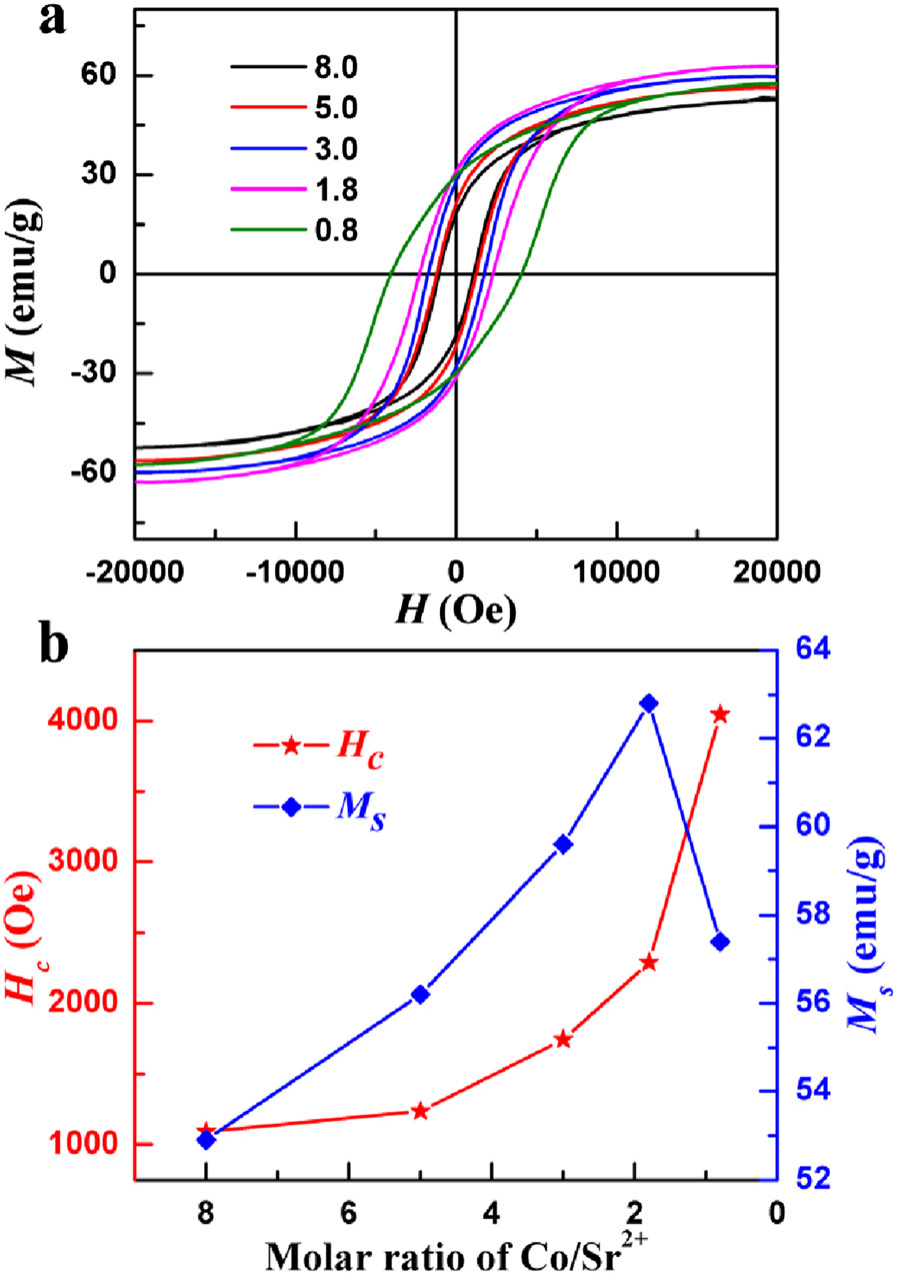
**Magnetic hysteresis loops and tendency of** ***M***_s_ **and** ***H***_c_ **of the samples. (a)** Magnetic hysteresis loops of the samples with different Co/Sr^2+^ molar ratios. **(b)** The tendency of *M*_s_ and *H*_c_ of the samples with different Co/Sr^2+^ molar ratios.

Figure 5a shows the magnetic hysteresis loops of the nanofibers measured with a VSM at room temperature. The results demonstrate a good single-phase magnetic behavior, and the magnetization changes smoothly with the applied field, which suggest that the soft and hard magnetic phase exchange coupled strongly [8,25].

The tendency of *M*_s_ and *H*_c_ of the samples versus Co/Sr^2+^ molar ratios is illustrated in Figure 5b. It is observed that the *M*_s_ increases monotonously and reaches to the maximum value of 62.8 emu/g corresponding to the sample with the Co/Sr^2+^ molar ratio of 1.8. Subsequently, the *M*_s_ decrease drastically with the further decrease of the Co/Sr^2+^ molar ratios. The corresponding *H*_c_ values increase monotonously with the decrease of the Co/Sr^2+^ molar ratios in Figure 5b. This may be due to the fact that SrFe_12_O_19_ possesses a bigger magnetocrystalline anisotropy, and the magnetizations are mainly determined by the exchange coupling interaction between the soft and hard phases with increasing Sr^2+^ [26]. And the parameters of the remanent magnetization *M*_r_, squareness *M*_r_/*M*_s_, and the energy product of the nanofibers are shown in Table 2.

**Table 2:** **The part parameters of magnetic performances of CoFe**_2_ **O**_4_ **/SrFe**_12_ **O**_19_ **nanocomposite nanofibers**

**Co/Sr molar ratio**	***M***_s_ **(emu/g)**	***M***_r_ **(emu/g)**	***M***_r_ **/** ***M***_s_	**(BH)**_max_ **(MG Oe)**
8.0	52.90	18.22	0.34	0.36
5.0	56.20	21.50	0.38	0.47
3.0	59.6	28.10	0.47	0.91
1.8	62.8	31.21	0.50	1.29
0.8	57.4	29.80	0.52	2.20

It is well known that the exchange coupling interaction and dipolar interaction play a major role to determine the magnetic properties of the magnetic nanocomposite materials. There exist three different models of exchange coupling interaction formed at the interface between soft-soft, hard-hard, and soft-hard grains, which has been proposed by Han et al. in 2004 [27]. The sufficient exchange coupling will not only arrange the magnetization in the CoFe_2_O_4_ grains but also make the magnetic moments of the interface of the CoFe_2_O_4_/SrFe_12_O_19_ nanocomposite deviating from the local easy axis and aligning in parallel with each other, which leads to a higher value of the *M*_s_ [28]. The coercivity of the samples increased from 1,089 to 4,046 Oe continuously, which is owing to the high *H*_*k*_ of SrFe_12_O_19_ nanofibers. And with increasing ratio of SrFe_12_O_19_, the exchange coupling interactions between the hard/soft magnetic phases are enhanced, and the *H*_c_ and *M*_s_ all increased.

Simultaneously, the influence of crystallite size on the *H*_c_ and *M*_s_ is displayed as follows. The theoretical calculation suggests that the critical dimension of the soft phase (*t*_s_) in the hard/soft composite materials should be less than twice of the domain wall width (*δ*_ω_) of the hard phase for a perfect demagnetization of hard/soft phases [29], and the domain wall width of the SrFe_12_O_19_ is approximately 9 nm [30]. And another theoretical calculation indicates that the critical size of both phases should be about equal [31]. An optimal value of the crystallite size of the hard and soft phases results in the maximum of *M*_s_. While the value of the Co/Sr^2+^ was 0.8, the average crystallite size of the CoFe_2_O_4_ and SrFe_12_O_19_ has a wide difference, which leads to a decrease in *M*_s_. The comparison of the magnetic properties, morphologies, and annealing temperature between the results of the CoFe_2_O_4_/SrFe_12_O_19_ nanocomposites is published by previous literatures. The higher *M*_s_ was obtained in this work in a lower annealing temperature. The *H*_c_ was moderate because of lower Sr^2+^ content compared with other articles. The details of parameters of performances are listed in Table 3.

**Table 3:** **The comparison of the properties between the CoFe**_2_ **O**_4_ **/SrFe**_12_ **O**_19_ **nanocomposites prepared by electrospinning and other methods**

**Preparation method**	***M***_s_ **(emu/g)**	***H***_c_ **(Oe)**	**Structure**	**Annealing temperature**	**Reference**
Coprecipitation	27.9	3,567	Core-shell nanoparticle	800°C	[15]
Modified flux method	50	1,798	Nanoparticle	900°C	[14]
Coprecipitation	42.5	2,475	Nanoparticle	1,000°C	[16]
Electrospinning	60.9	3,190	Nanofibers	900°C	Our group [9]
Electrospinning	62.8	2,290	Nanofibers	800°C	This work

## Conclusions

CoFe_2_O_4_/SrFe_12_O_19_ nanofibers were fabricated by electrospinning and calcination process. Simultaneously, a novel way that cobalt powder was used in the precursor sol-gel instead of cobalt salt was adopted in this work. What is more, the samples had an enhanced saturation magnetization of 62.8 emu/g and coercivity of 2,290 Oe when the average crystallite size of the hard/soft phases reached up to an optimal value. And the hard/soft magnetic nanocomposite exhibited a strong exchange coupling interaction.
